# Reduced Bone Mass and Muscle Strength in Male 5α-Reductase Type 1 Inactivated Mice

**DOI:** 10.1371/journal.pone.0021402

**Published:** 2011-06-22

**Authors:** Sara H. Windahl, Niklas Andersson, Anna E. Börjesson, Charlotte Swanson, Johan Svensson, Sofia Movérare-Skrtic, Klara Sjögren, Ruijin Shao, Marie K. Lagerquist, Claes Ohlsson

**Affiliations:** 1 Centre for Bone and Arthritis Research, Institute of Medicine, Sahlgrenska Academy at University of Gothenburg, Gothenburg, Sweden; 2 Institute of Neuroscience and Physiology, Sahlgrenska Academy at University of Gothenburg, Gothenburg, Sweden; Institut de Génomique Fonctionnelle de Lyon, France

## Abstract

Androgens are important regulators of bone mass but the relative importance of testosterone (T) *versus* dihydrotestosterone (DHT) for the activation of the androgen receptor (AR) in bone is unknown. 5α-reductase is responsible for the irreversible conversion of T to the more potent AR activator DHT. There are two well established isoenzymes of 5α-reductase (type 1 and type 2), encoded by separate genes (*Srd5a1* and *Srd5a2*). 5α-reductase type 2 is predominantly expressed in male reproductive tissues whereas 5α-reductase type 1 is highly expressed in liver and moderately expressed in several other tissues including bone. The aim of the present study was to investigate the role of 5α-reductase type 1 for bone mass using *Srd5a1^−/−^* mice. Four-month-old male *Srd5a1* ^−/−^ mice had reduced trabecular bone mineral density (−36%, p<0.05) and cortical bone mineral content (−15%, p<0.05) but unchanged serum androgen levels compared with wild type (WT) mice. The cortical bone dimensions were reduced in the male *Srd5a1* ^−/−^ mice as a result of a reduced cortical periosteal circumference compared with WT mice. T treatment increased the cortical periosteal circumference (p<0.05) in orchidectomized WT mice but not in orchidectomized *Srd5a1* ^−/−^ mice. Male *Srd5a1* ^−/−^ mice demonstrated a reduced forelimb muscle grip strength compared with WT mice (p<0.05). Female *Srd5a1* ^−/−^ mice had slightly increased cortical bone mass associated with elevated circulating levels of androgens. In conclusion, 5α-reductase type 1 inactivated male mice have reduced bone mass and forelimb muscle grip strength and we propose that these effects are due to lack of 5α-reductase type 1 expression in bone and muscle. In contrast, the increased cortical bone mass in female *Srd5a1* ^−/−^ mice, is an indirect effect mediated by elevated circulating androgen levels.

## Introduction

Androgens are of major importance for bone growth and maintenance [1]–[3]. The effects of testosterone (T) can be exerted either directly through the androgen receptor (AR) or indirectly through aromatization to estrogens, exerting their effects through estrogen receptors (ERα and/or ERβ [1], [2]). All these receptors are expressed both in growth plate cartilage and in bone [4]–[8]. Both ERα-activation and AR-activation but not ERβ-activation are required for a normal bone mass and bone health in males [9]–[14]. 5α-reductase enzymes are responsible for the irreversible conversion of T to the more potent AR activator DHT. We have previously reported that DHT treatment increases bone mass in orchidectomized mice and that this effect is independent of estrogen receptors [11]. The relative importance of endogenous T *versus* DHT for the activation of the AR in bone is unknown.

The DHT-AR complex has a longer half-life and a higher DNA binding affinity than the T-AR complex. Therefore, the effective dose of DHT, required to activate an androgen responsive marker gene by 50%, is about 10-fold lower than that required to achieve the same level of induction with T [15]–[19]. It is proposed that the conversion of T to DHT by tissue specific 5α-reductase activity results in a tissue specific signal amplification of the androgenic activity [20].

There are two well established isoenzymes of 5α-reductase (type 1 and type 2), encoded by separate genes (*Srd5a1* and *Srd5a2*; [21]–[25]). In addition, it has recently been proposed by Uemeura *et al* that a third 5α-reductase enzyme, type 3 encoded by *Srd5a3*, has the capacity to produce DHT from T [25]. However, others claim that 5α-reductase type 3 has little or no functional ability to reduce steroid substrates but rather has a crucial role in N-linked protein glycosylation [21], [24].

Both 5α-reductase type 1 and type 2 clearly have the capacity to convert T to DHT but they have different tissue distribution and enzymatic activities. 5α-reductase enzymes have the capacity to catalyse not only anabolic but also catabolic reactions in the androgen metabolism [26]. Thus, conversion of T into DHT also marks the latter hormone for degradation to the inactive compound 3α-Adiol [27]. The biochemical properties and tissue distribution of the type 1 isoenzyme are those of a catabolic agent, with a low affinity for steroid substrates and a high expression in the liver. In contrast, a high substrate affinity and a predominant expression in male reproductive tissues indicate that the type 2 isoenzyme is an anabolic entity [26]. However, 5α-reductase type 1 is not only expressed in the liver but also in several peripheral tissues including bone and cartilage, suggesting that it might be involved in signal amplification of the androgenic activity in the skeleton [28]–[30].

There are no reported cases of 5α-reductase type 1 deficiency in humans. Female *Srd5a1* ^−/−^ mice exhibit a parturition defect due to impaired cervical ripening caused by a defected local catabolism of progesterone [31], while male mice have an apparent normal reproductive phenotype [27].

Since 5α-reductase type 1 is expressed in bone and has the capacity to amplify the androgenic signal, we hypothesized that 5α-reductase type 1 mediated local conversion of T to the more potent androgen DHT might be of importance for bone metabolism. We, therefore, analyzed the skeletal phenotype of *Srd5a1* ^−/−^ mice.

## Results

### Tissue distribution of 5α-reductase isoforms

**Figure 1:**
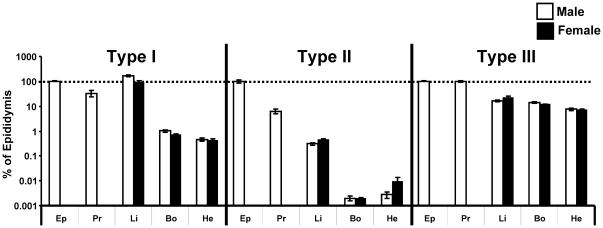
Tissue distribution of 5α-reductase type 1–3. Real-time PCR showing the relative mRNA levels of *Srd5a1* (type 1), *Srd5a2* (type 2), and *Srd5a3* (type 3) in epididymis (Ep), prostate (Pr), liver (Li), bone (Bo) and heart (He). Values are given as % of levels in epididymis and are means ± SEM (males n = 5, females n = 5).

The mRNA levels of the two well established 5α-reductase isoforms, type 1 and type 2, were analyzed in several tissues from adult wild type (WT) mice. The highest mRNA levels of 5α-reductase type 1 were found in liver, while the highest levels of 5α-reductase type 2 were found in male reproductive tissues (epididymis and prostate; Fig. 1). The type 1 mRNA levels were ≅100-fold while the type 2 mRNA levels were as much as ≅50.000-fold lower in bone compared with the tissue with the highest expression of the respective 5α-reductase isoform (Fig. 1). In addition, we analyzed the tissue distribution of the proposed, but less established, 5α-reductase type 3 enzyme, demonstrating the highest mRNA levels in male reproductive tissues and ≅10-fold lower mRNA levels in liver and bone (Fig. 1). The general tissue distribution of the three 5α-reductase isoforms in non-reproductive tissues did not differ between male and female mice.

### Body weight and visceral organs in *Srd5a1^−/−^* mice

There was no significant difference in body weight, crown-rump length or tibia length between *Srd5a1* ^−/−^ and WT mice (Table 1). Furthermore, *Srd5a1* ^−/−^ mice displayed normal weights of seminal vesicles, uterus and gonadal fat. Female but not male *Srd5a1* ^−/−^ mice had reduced thymus weight compared with WT mice (Table 1).

**Table 1:** Body and organ weights.

	Male		Female	
	WT (n = 7)	KO (n = 8)	WT (n = 13)	KO (n = 8)
Body weight (g)	34.2±0.8	31.6±1.4	25.6±0.6	26.9±1.9
Crown-rump length (mm)	60.5±0.4	59.4±0.5	56.8±0.6	57.3±1.0
Tibia length (mm)	19.0±0.2	18.8±0.2	18.4±0.2	18.5±0.2
Seminal vesicle weight/BW (mg/g)	7.9±0.4	8.1±0.8	NA	NA
Uterus weight/BW (mg/g)	NA	NA	5.6±0.5	4.2±0.4
Gonadal fat weight/BW (mg/g)	33.6±2.8	32.5±3.3	51.9±3.1	45.5±5.1
Thymus weight/BW (mg/g)	1.6±0.1	1.5±0.1	2.5±0.2	2.0±0.1*
Liver weight/BW (mg/g)	35.2±1.7	36.2±1.3	35.8±1.5	37.1±1.5
*M. tibialis* weight/BW (mg/g)	1.31±0.07	1.22±0.07	1.15±0.04	1.18±0.06
*M. quadriceps* weight/BW (mg/g)	6.19±0.25	5.95±0.37	5.56±0.20	5.45±0.34

Four-month-old *Srd5α1* ^−/−^ (KO) and wild type (WT) mice. Values are means ± SEM.

*p<0.05 Student's t-test *versus* WT, NA = not applicable, BW = body weight, M = Muscle.

### Reduced areal BMD and BMC in *Srd5a1^−/−^* mice

**Figure 2:**
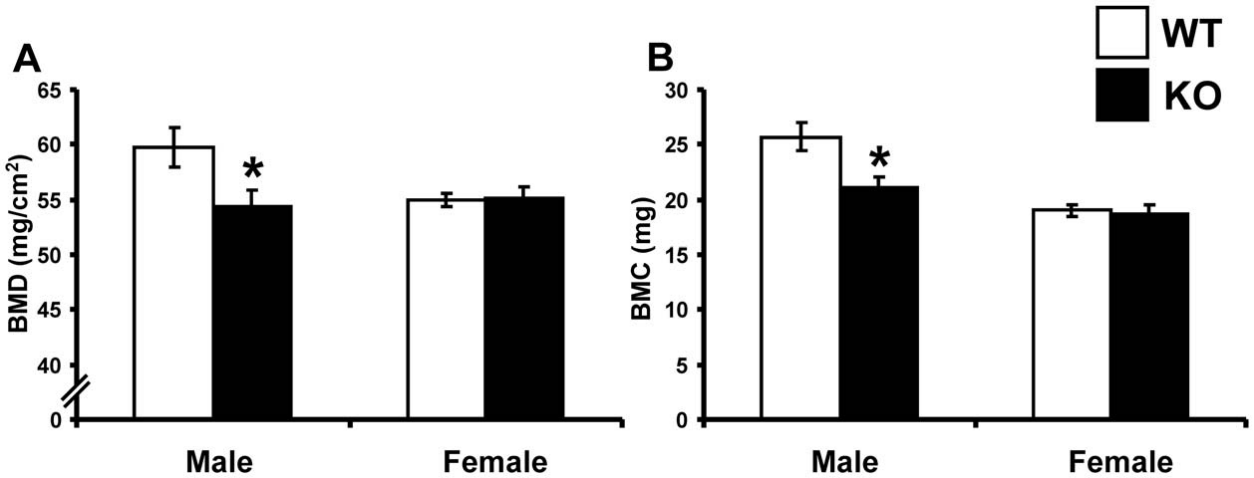
Reduced areal BMD and BMC in male *Srd5α1^−/−^* mice. Areal bone mineral density (BMD; A) and bone mineral content (BMC; B) of tibia as analyzed by DXA in four-month-old *Srd5α1^−/−^* (KO; males n = 8, females n = 8) and wild type (WT; males n = 7, females n = 13) mice. Values are given as means ± SEM. * p<0.05 Student's t-test *versus* WT.

Dual energy X-ray absorptiometry (DXA) measurements of tibia demonstrated that the areal BMD (−8.9%, p<0.05) and the BMC (−17.8%, p<0.01) were reduced in four-month-old male *Srd5a1* ^−/−^ mice compared with male WT mice (Fig. 2). Neither areal BMD nor BMC was significantly affected in female *Srd5a1* ^−/−^ mice compared to female WT mice.

### Reduced trabecular BMD and cortical BMC in male, but increased cortical BMC in female *Srd5a1* ^−/−^ mice

**Figure 3:**
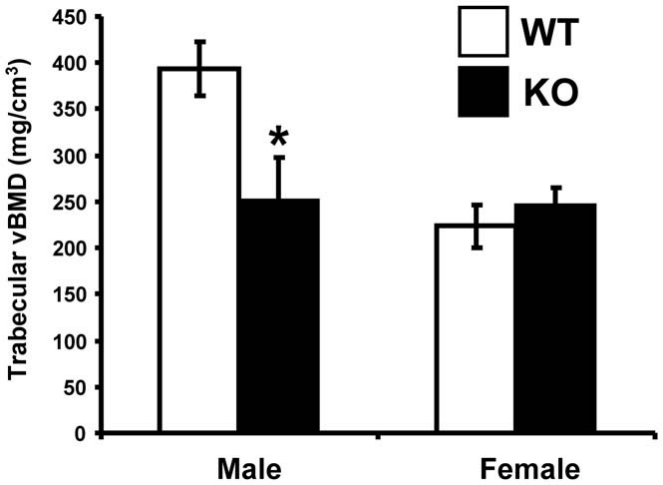
Reduced trabecular BMD in male *Srd5α1* ^−/−^ mice. Trabecular bone mineral density (BMD) as analyzed by pQCT in the distal metaphyseal region of femur in four-month-old *Srd5α1* ^−/−^ (KO; males n = 8, females n = 8) and wild type (WT; males n = 7, females n = 12) mice. Values are given as means ± SEM. * p<0.05 Student's t-test *versus* WT.

**Figure 4:**
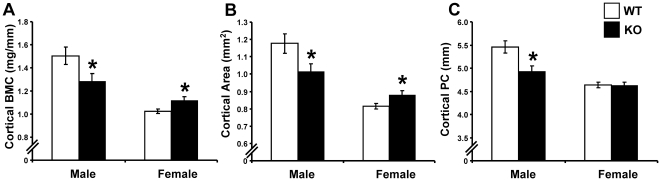
Cortical bone parameters in *Srd5α1^−/−^* mice. Cortical bone mineral content (BMC; A), area (B) and periosteal circumference (PC; C) as analyzed by pQCT in the mid diaphyseal region of femur in four-month-old *Srd5α1* ^−/−^ (KO; males n = 8, females n = 8) and wild type (WT; males n = 7, females n = 12) mice. Values are given as means ± SEM. * p<0.05 Student's t-test *versus* WT.

As the DXA technique cannot distinguish between the cortical and trabecular bone compartments, detailed analyses using pQCT were performed to further characterize the bone phenotype in *Srd5a1* ^−/−^ mice. The trabecular BMD was reduced (−36%, p<0.05) in male *Srd5a1* ^−/−^ mice compared with WT mice while it was not significantly affected in female *Srd5a1* ^−/−^ mice (Fig. 3). Cortical bone parameters were determined with mid-diaphyseal pQCT scans of the long bones. The cortical bone mineral content (BMC) was decreased in male (−14.6%, p<0.05) but slightly increased in female (+9.1%, p<0.05) *Srd5a1* ^−/−^ mice compared with WT mice (Fig. 4A). The reduced cortical BMC in male *Srd5a1* ^−/−^ mice was due to a reduced cortical bone area (−13.9%; p<0.05) while the cortical BMD was unaffected compared with WT mice (Fig. 4B and Table 2). The cortical bone area, in turn, was reduced as a result of reduced periosteal circumference (Fig. 4C; −9.8%, p<0.05). Consequently, male *Srd5a1* ^−/−^ mice displayed a reduction of cortical bone parameters reflecting bone strength, including cortical cross-sectional moment of inertia and cortical moment of resistance (p<0.05; Table 2). The increased cortical BMC in female *Srd5a1* ^−/−^ mice was due to an increased cortical bone area (+7.9%; p<0.05) associated with an increased cortical thickness (+9.6%; p<0.05; Fig. 4B, Table 2).

**Table 2:** Cortical bone parameters.

	Male		Female	
	WT (n = 7)	KO (n = 8)	WT (n = 12)	KO (n = 8)
Cortical vBMD (mg/cm^3^)	1278±8	1266±9	1253±5	1268±8
Cortical thickness (µm)	252±9	243±6	204±2	224±3*
Endosteal circumference (mm)	3.88±0.13	3.40±0.1*	3.36±0.05	3.22±0.05
MR (mm^3^)	0.65±0.04	0.50±0.04*	0.39±0.01	0.42±0.02
MI (mm^4^)	0.68±0.06	0.46±0.05*	0.32±0.01	0.33±0.02

Cortical bone parameter as analyzed by pQCT in the mid diaphyseal region of femur in four-month-old *Srd5α1* ^−/−^ (KO) and wild type (WT) mice. vBMD = volumetric bone mineral density, MR = cortical cross sectional moment of resistance, MI = cortical cross sectional moment of inertia. Values are means ± SEM.

*p<0.05 Student's t-test *versus* WT.

As expected, male WT mice had higher trabecular BMD (+76.2%, p<0.05) and cortical periosteal circumference (+17.7%, p<0.05) than female WT mice. In contrast, no significant gender difference for these two parameters was seen in *Srd5a1* ^−/−^ mice (Fig. 3 and Fig. 4). Thus, the normal gender differences on trabecular BMD and outer dimensions of the cortical bone were lost in *Srd5a1* ^−/−^ mice.

Neither the trabecular BMD nor the cortical BMC were affected in one-month-old pre-pubertal *Srd5a1* ^−/−^ mice compared with WT mice (data not shown).

### Elevated androgen levels in female *Srd5a1* ^−/−^ mice

**Figure 5:**
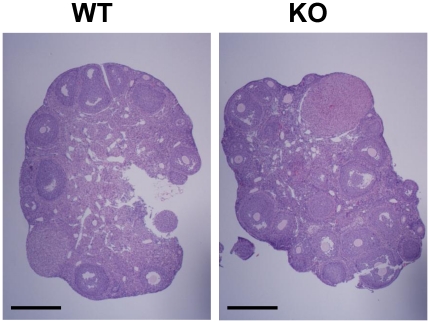
Ovarian morphology of WT and *Srd5a1* ^−/−^ (KO) mice. Hematoxylin and eosin stained ovarian sections from WT and KO mice. Bar scale: 500 µm.

Serum levels of androgens and estradiol were not affected in male *Srd5a1* ^−/−^ mice while both serum levels of T and DHT were increased in adult female *Srd5a1* ^−/−^ mice compared with WT mice (Table 3). To investigate if the increase in serum androgens in female *Srd5a1* ^−/−^ was due to a disturbed central negative feed-back regulation, serum levels of luteinizing hormone (LH) were analyzed. Serum LH levels were not increased but rather decreased in female *Srd5a1* ^−/−^ compared with WT mice, suggesting a normal central negative feed-back regulation of serum sex steroids in female *Srd5a1* ^−/−^ mice (Table 3). To address the possibility that the high serum androgens could be the result of a local disturbance in the ovaries associated with elevated androgen synthesis, the ovarian morphology and LH receptor (LHR) mRNA levels were analyzed. The ovaries in *Srd5a1* ^−/−^ mice had a normal morphology (Fig. 5), and the LHR mRNA levels were unaffected (WT 1.0±0.3 and *Srd5a1* ^−/−^ 1.1±0.2 LHR/GAPDH ratio; non-significant). Female *Srd5a1* ^−/−^ mice had unchanged serum estradiol levels (Table 3) and displayed a normal estrus cycle, with unaffected cycle length (WT 4.2±0.2 days, *Srd5a1* ^−/−^ 4.3±0.3 days; non-significant).

**Table 3:** Serum levels of hormones and mRNA levels of major urinary protein and prolactin receptor in liver.

	Male		Female	
	WT (n = 7)	KO (n = 8)	WT (n = 13)	KO (n = 8)
Estradiol (pg/ml)	8.5±1.0	9.6±1.2	14.5±1.1	13.8±1.2
Testosterone (ng/ml)	6.7±1.2	6.5±1.1	ND	0.43±0.24*
DHT (ng/ml)	1.59±0.33	1.48±0.28	0.14±0.02	0.41±0.10*
LH (ng/ml)	NA	NA	0.25±0.05	0.11±0.03*
IGF-1 (ng/ml)	200±12	220±14	174±13	188±19
*Mup*/GAPDH ratio	2.30±0.48	2.21±0.49	0.12±0.02	0.16±0.06
*Prlr*/GAPDH ratio	0.71±0.21	0.49±0.15	2.06±0.24	2.44±0.51

Serum hormone levels and relative amounts of liver major urinary protein (*mup*) and prolactin receptor (*Prlr*) mRNA were measured in four-month-old *Srd5α1* ^−/−^ (KO) and wild type (WT) mice. Values are given as means ± SEM.

*p<0.05 vs. wild-type (WT), NA = not available, ND = not detectable, DHT = dihydrotestosterone, LH = luteinizing hormone, IGF-I = insulin like growth factor 1.

To evaluate if the partial feminization of the male skeleton was due to alterations in serum insulin like growth factor 1 (IGF-I) and/or the growth hormone (GH)-secretion pattern, serum levels of IGF-1 as well as major urinary protein (MUP; high levels reflecting a male GH-secretion pattern; [32]) and prolactin receptor (high levels reflecting a female GH-secretion pattern) mRNA levels in the liver were measured. As expected, WT males had higher MUP and lower prolactin receptor mRNA levels in the liver compared to female mice. However, serum IGF-I, MUP mRNA and prolactin receptor mRNA levels were not affected in *Srd5a1* ^−/−^ mice compared with WT mice (Table 3).

### Reduced effect of testosterone on cortical bone parameters in orchidectomized *Srd5a1* ^−/−^ mice

**Figure 6:**
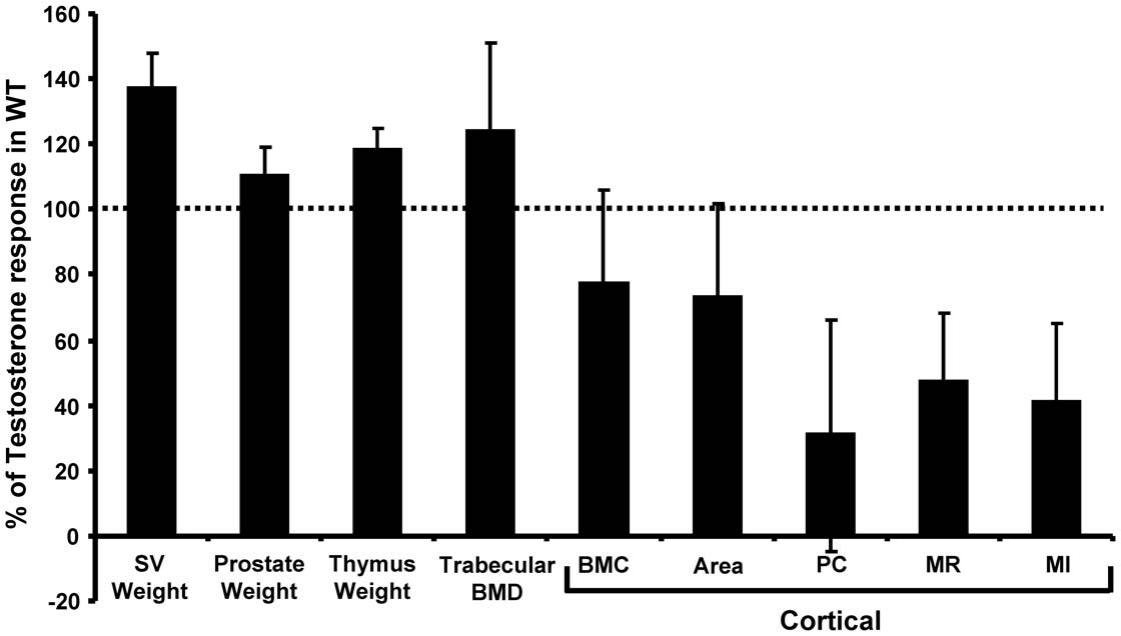
Tissue dependent testosterone response in orchidectomized *Srd5α1^−/−^* mice. Four-month-old orchidectomized mice were either treated with placebo or testosterone (1.5 mg/60days) for four weeks. The bars represent the testosterone response in percent for the *Srd5α1* ^−/−^ mice compared to the testosterone response in WT mice. Thus, 0% means no testosterone response while 100% is a normal WT testosterone response (WT orx n = 12, WT orx+T n = 10, KO orx n = 8, KO orx+T n = 8). Values are given as means ± SEM. Weights of seminal vesicle (SV), prostate and thymus were adjusted for body weight. BMC = bone mineral content, BMD = bone mineral density, PC = periosteal circumference, MR = cortical cross sectional moment of resistance, MI = cortical cross sectional moment of inertia. Values are given as means ± SEM.

To determine if the responses to T treatment were affected by 5α-reductase inactivation, orchidectomized (orx) *Srd5a1* ^−/−^ mice and orx WT mice were treated with T or placebo for four weeks. As expected, weights of seminal vesicles and prostate, trabecular BMD, cortical BMC and cortical periosteal circumference were increased, while the weight of thymus was decreased by T treatment in orx WT mice (Table 4). The significant effects of T on prostate weight, trabecular BMD and thymus weight were similar in orx *Srd5a1* ^−/−^ mice compared with orx WT. In contrast, no significant effect of T was seen on the cortical bone parameters in orx *Srd5a1* ^−/−^ mice (Table 4, Fig. 6).

**Table 4:** Effect of T treatment in orchidectomized mice.

	WT(% over orx placebo)	KO(% over orx placebo)
Seminal vesicles weight/BW	1028±58*	1413±111*
Prostate weight/BW	390±29*	430±37*
Thymus weight/BW	−62±4*	−73±4*
Trabecular BMD	110±16*	136±29*
Cortical BMC	16±3*	12±4
Cortical area	13±2*	10±4
Cortical PC	4.9±1,4*	1.5±1,7
Cortical EC	2.8±1,8	−3.0±2,3
Cortical MR	27±5*	12±6
Cortical MI	29±7*	12±7

Four-month-old orchidectomized (orx) mice were either treated with placebo or testosterone (1.5 mg/60days) for four weeks. The effect of testosterone is given as the testosterone treated group expressed as percent over the placebo treated group in *Srd5α1* ^−/−^ (KO) and wild type (WT) mice (WT orx n = 12, WT orx+T n = 10, KO orx n = 8, KO orx+T n = 8). Values are means ± SEM. BW = body weight, BMC = bone mineral content, BMD = bone mineral density, PC = periosteal circumference, EC = endosteal circumference, MR = cross sectional moment of resistance, MI = cross sectional moment of inertia.

*p<0.05 testosterone treated *versus* orx placebo.

### Reduced forelimb muscle grip strength in *Srd5a1* ^−/−^ mice

**Figure 7:**
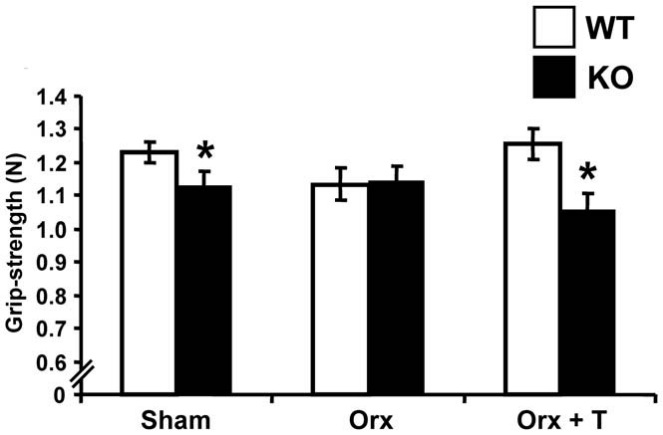
Reduced forelimb grip strength in male *Srd5α1* ^−/−^ mice. Grip strength in four-month-old gonadal intact (sham-operated) *Srd5α1* ^−/−^ (KO; n = 18) and wild type (WT; n = 27) mice. In addition, four-month-old orchidectomized (orx) KO and WT mice were either treated with placebo (Orx) or testosterone (Orx+T) for four weeks (WT orx n = 13, WT orx+T n = 13, KO orx n = 8, KO orx+T n = 8). Values are means ± SEM. * p<0.05 Student's t-test *versus* WT.

The weights of the *tibialis* and *quadriceps* muscles were not significantly affected in *Srd5a1* ^−/−^ mice compared with WT mice (Table 1). Interestingly, male *Srd5a1* ^−/−^ mice had reduced forelimb muscle strength compared with WT mice (Fig. 7). In addition, T treated but not placebo treated orx *Srd5a1* ^−/−^ mice had reduced forelimb muscle grip strength compared with corresponding WT mice (Fig. 7). Thus, in the presence of either endogenous or exogenous T, but not in the T deficient state, male *Srd5a1* ^−/−^ mice demonstrated a reduced grip strength.

## Discussion

Androgens are important regulators of bone mass, but the relative importance of T *versus* DHT for the activation of the AR in bone is unknown. Using *Srd5a1^−/−^* mice, we herein provide evidence that 5α-reductase type 1 mediated conversion of T to DHT in the target tissue results in a signal amplification of the androgenic activity on bone mass and muscle strength without affecting circulating androgen levels in male mice. In contrast, in female mice 5α-reductase type 1 is mainly involved in the degradation of androgens, supported by elevated circulating androgen levels in female *Srd5a1^−/−^* mice.

The main finding of the present study was that male *Srd5a1* ^−/−^ mice had reduced bone mass although their circulating androgen levels were unaffected. DXA analysis demonstrated that male *Srd5a1* ^−/−^ mice had reduced areal BMD and further pQCT analysis revealed that it was caused by a combination of reduced trabecular BMD and cortical bone size compared with WT mice. This bone phenotype resembles the phenotype seen in partial T deficient male mice [2] suggesting that it was caused by a lack of a local 5α-reductase type 1 dependent amplification of the androgenic signaling by conversion of T to the more potent androgen DHT locally in bone. It is a limitation with the present study that we did not treat the *Srd5a1* ^−/−^ mice with DHT as it would have been a definitive proof that these KO mice can respond to DHT and it might have provided some insight on the relative importance of circulating vs. locally produced DHT. A specific role of 5α-reductase type 1 in bone is supported by our finding that the response to T treatment in orx *Srd5a1* ^−/−^ mice was reduced in cortical bone but not in some other T responsive tissues.

The normal gender difference with larger outer dimensions of the cortical bone in males *versus* females was lost in the *Srd5a1* ^−/−^ mice. The sexual dimorphism of the skeleton has been linked to gender differences in the GH-secretion pattern [32] but parameters reflecting gender specific GH-secretion were not affected in the *Srd5a1* ^−/−^ mice compared with WT mice, supporting the notion that the skeletal phenotype in male *Srd5a1* ^−/−^ mice was due to lack of 5α-reductase type 1 expression in bone.

A role of 5α-reductase type 1 in bone is supported by previous studies showing that 5α-reductase activity is present in human bone [29] and 5α-reductase type 1 is expressed in cultured osteoblasts [28]. In addition, in the present study we demonstrated that 5α-reductase type 1 is clearly expressed while 5α-reductase type 2 is barely expressed in mouse bone. Our findings suggest that for optimal bone health it is beneficial to use 5α-reductase type 2 specific inhibitors (such as finasteride) and not combined type 2 and type 1 inhibitors in the treatment of patients with benign prostatic hyperplasia and/or androgenetic alopecia.

Both muscle mass and strength have been associated with bone mass and androgens are important regulators of both muscle mass and strength [13], [33]. Muscle mass was not significantly affected in *Srd5a1* ^−/−^ mice, but interestingly, male *Srd5a1* ^−/−^ mice had reduced forelimb muscle strength, suggesting that local 5α-reductase type 1 expression is of importance for the signal amplification of the androgenic activity not only in bone but also in muscle. As exogenous testosterone treatment has been shown to increase both muscle mass and muscle strength it is somewhat unexpected that grip strength but not muscle mass was significantly reduced in the male *Srd5a1* ^−/−^ mice [34]. However, one cannot exclude that the lack of significant effect on muscle mass was due to the relatively small number of mice evaluated. Our findings may suggest that 5α-reductase type 1 specific enhancers could be useful for the treatment of men with osteoporosis and/or sarcopenia.

The total body weight and the weights of several visceral organs, including androgen sensitive organs such as seminal vesicles and thymus were normal in male *Srd5a1* ^−/−^ mice, indicating that the effects seen on bone mass and muscle strength are tissue-specific in males.

The phenotype of the female *Srd5a1* ^−/−^ mice is clearly confounded by the elevated circulating levels of androgens, which most probably caused the increased cortical bone mass and reduced thymus weight. These two effects are well-established to be caused by supra-physiological androgen treatment [11], [12], [35], [36]. Not only serum T but also serum DHT levels were elevated in the female *Srd5a1* ^−/−^ mice, demonstrating that 5α-reductase type 1 is not crucial for the conversion of T to DHT in female mice. The elevated serum androgen levels were not the result of a disturbed negative feed-back regulation, supported by the finding that serum LH was not elevated in female *Srd5a1* ^−/−^ mice. Female *Srd5a1* ^−/−^ mice displayed a normal ovarian morphology and LH receptor mRNA levels and a normal estrus cycle, with unaffected cycle length, indicating that there was no major ovarian dysfunction leading to increased ovarian T production. The high expression of 5α-reductase type 1 in liver in WT mice together with the elevation of circulating androgen levels in female *Srd5a1* ^−/−^ mice suggest that the elevated serum androgen levels were a result of the lack of 5α-reductase type 1 dependent inactivation/degradation of androgens in the liver of female *Srd5a1* ^−/−^ mice. An important role of 5α-reductase type 1 for androgen degradation in females is supported by several previous studies [27], [31], [37], [38]. No elevation of serum androgen levels was seen in male *Srd5a1* ^−/−^ mice which could be explained by the high expression of 5α-reductase type 2, normally seen in male reproductive tissues, having the capacity to replace the 5α-reductase type 1 enzyme in androgen degradation in male *Srd5a1* ^−/−^ mice.

Since the female *Srd5a1* ^−/−^ mice had both increased circulating levels of androgens and a deletion of *Srd5a1* ^−/−^, the physiological role of *Srd5a1* ^−/−^ expression in bone cannot be evaluated by using the female *Srd5a1* ^−/−^ mouse model. However, the female *Srd5a1* ^−/−^ mouse model could be useful for studies evaluating the role of chronic elevation of circulating androgen levels on for instance metabolism and cardiovascular health. Interestingly, genetic variants in the *Srd5a1* gene have been associated with two androgen linked conditions, polycystic ovary syndrome and hirsutism, in females [38].

In conclusion, 5α-reductase type 1 inactivated male mice have reduced bone mass and forelimb muscle grip strength and we propose that these effects might be direct due to lack of 5α-reductase type 1 expression in bone and muscle. In contrast, the increased cortical bone mass in female *Srd5a1* ^−/−^ mice is an indirect effect caused by elevated circulating androgen levels, which in turn probably is a consequence of 5α-reductase type 1 expression being rate-limiting for androgen degradation in female mice.

## Materials and Methods

### Animals

Animal care was in accordance with institutional guidelines and the study protocol was approved by the local ethical committee (permit number 362-2005 and 182-2006). All efforts were made to minimize suffering. The mice were housed in a standard animal facility under controlled temperature (22°C) and photoperiod (12 h of light, 12 h of dark) with free access to water and standard food. *Srd5a1^−^* ^/−^ mice were developed as previously described [27]. *Srd5a1* ^−/−^ and littermate WT mice were analyzed at 1 (pre-pubertal) and 4 (adult) months of age. In addition, four-month-old orchidectomized (orx) *Srd5a1* ^−/−^ and WT mice were either treated with placebo or T (1.5 mg/60days) for four weeks using slow release pellets (Innovative Research of America, Sarasota, FL, USA), delivering a physiological dose of T or placebo. Initial detailed dose-response studies of the effect of T established a dose of 1.5 mg/60 days to normalize the weight of the seminal vesicles in orx WT mice.

### Dual X-ray absorption analysis

Areal bone mineral density and bone mineral content of excised tibia were measured by DXA using the Lunar PIXImus Mouse Densitometer (Wipro GE Healthcare, Madison, WI).

### Peripheral Quantitative Computerized Tomography analyses

Computerized tomography was performed with the STRATEC pQCT XCT, operating at a resolution of 70 µm as previously described [14]. Trabecular BMD was determined *ex vivo*, with a metaphyseal pQCT scan of the distal femur. The scan was positioned in the metaphysis at a distance from the distal growth plate corresponding to 4% of the total length of the femur, and the trabecular bone region was defined as the inner 45% of the total cross-sectional area. Cortical bone parameters were determined *ex vivo* with a mid-diaphyseal pQCT scan of the long bones [39].

### Serum analyses

Serum analyses were performed according to the manufacturer's instructions for estradiol (Estradiol ELISA, Bioquant, San Diego, CA, sensitivity 5 pg/ml, intra-assay variation 10.3%), T (RIA, MP Biomedicals /ICN Biomedicals, Costa Mesa, CA, sensitivity 0.1 ng/ml, intra-assay variation 9.1%), DHT (ELISA kit from Alpha diagnostic International, San Antonio, TX, sensitivity 25 pg/ml, intra-assay variation 10.5%), luteinizing hormone (RIA AHR002, IDS Biocode-Hycel, Liege, Belgium, sensitivity 0.14 ng/ml, intra-assay variation 10.5%) and insulin like growth factor-1 (RIA, Mediagnost, Reutingen, Germany, sensitivity 0,020 ng/ml, intra-assay variation <3%).

### Ovarian analyses and vaginal smears

Ovaries were fixed in a 4% formaldehyde neutral-buffered solution for 24 h at 4°C and embedded in paraffin and sectioned in 5 µm sections for histological analyses. Ovarian sections were deparaffinised, rehydrated and stained with hematoxylin and eosin. The stage of cyclicity was determined by microscopic analysis of the predominant cell type in vaginal smears obtained daily during two cycles [40]. The vaginal smears were stained with hematoxylin and eosin using standard protocols and staged by light microscopy. Care was taken to avoid mechanical stimulation of the cervix during this procedure to prevent pseudopregnancy.

### Real-time PCR

RNA was prepared from liver, heart, prostate and epididymis using RNeasy kit (#74106, Qiagen, Hilden, Germany) according to the manufacturer's instructions. Total RNA from humerus was prepared using TriZol Reagent (Invitrogen, Lidingö, Sweden). The RNA was reverse transcribed into cDNA using High-capacity cDNA Reverse transcription kit (#4368814, Applied Biosystems, Stockholm, Sweden). Real-time PCR (RT-PCR) analyses were performed using the ABI Prism 7000 Sequence Detection System (PE Applied Biosystems, Carlsbad, CA). We used pre-designed RT-PCR assays from Applied Biosystems for the analysis of Srd5a1 (Mm00614213_ml), Srd5a2 (Mm01237407_ml), Srd5a3 (Mm00491099_m1), prolactin receptor (Mm00599957_m1), LH receptor (Mm00442931_m1) and GAPDH (internal control, 4352339E) mRNA levels (Applied Biosystems). For analysis of major urinary protein (MUP), the following forward primer MUP-1FP 5′GCT GCT GTG TTT GGG ACT GA, reverse primer MUP-1RP 5′AAG TTC CTT CCC GTA GAA CTA GCTT and probe MUP-1 MGB-Probe 5′CCT AGT CTG TGT CCA TGCA were used.

### Forelimb muscle grip strength

The forelimb muscle grip strength was determined using an automated grip strength meter (Columbus Instruments, Columbus, OH). Mice were lifted by their tail and were made to hold a horizontal bar with their forelimbs. Then, the mice were pulled backwards until they could no longer hold the grip. Maximal force was registered during consecutive attempts (four attempts/mouse for sham mice and eight attempts/mouse for orx mice) and the result was set as the average of the four best attempts. The analysis was performed in a blinded fashion.
